# Supplementary material for: Early Gestational Exposure to High-Molecular-Weight Phthalates and Its Association with 48-Month-Old Children’s Motor and Cognitive Scores
Source: Int J Environ Res Public Health. 2020 Nov 4;17(21):8150. doi: 10.3390/ijerph17218150 (PMC7662459; doi:10.3390/ijerph17218150)
Supplement: Supplementary file 1 [file ijerph-17-08150-s001.pdf]

SUPPLEMENTARY MATERIALS:

Table S1. Linear associations between urinary phthalate metabolites pregnancy geometric mean and McCarthy Scales of Children's Abilities

Metabolite	Motor Scale ß† (95% CI)	General Cognitive Index ß† (95% CI)	Memory Scale ß† (95% CI)
**∑ HMWP[1]**	-2.8 (-5.1, -0.5)*	-2.8 (-6.0, 0.4)	0.0 (-1.6, 1.7)
**MEHP**	-1.5 (-3.5, 0.5)	-1.2 (-4.0, 1.5)	0.7 (-0.7, 2.1)
**MEHHP**	-2.4 (-4.3, -0.4)*	-2.2 (-4.9, 0.5)	0.1 (-1.3, 1.5)
**MEOHP**	-2.3 (-4.3, -0.4*	-2.4 (-5.2, 0.3)	0.0 (-1.5, 1.4)
**MECPP**	-2.8 (-5.1, -0.5)*	-2.5 (-5.7, 0.6)	0.0 (-1.7, 1.7)
**MBzP**	-1.3 (-2.9, 0.3)	-2.0 (-4.2, 0.2)	-0.4 (-1.6, 0.8)
**MCPP**	-0.9 (-3.1, 1.2)	-1.3 (-4.2, 1.7)	-0.3 (-1.8, 1.3)
**∑ DEHP[2]**	-2.8 (-5.0, -0.5)*	-2.6 (-5.8, 0.5)	0.1 (-1.5, 1.7)

† Adjusted for sex, gestational age, breastfeeding and maternal IQ
1 High Molecular Weight Phthalate Molar Sum: MEHP, MEHHP, MEOHP, MECPP, MBzP and MCPP.
2 Molar Sum of the four metabolites derived from DEHP: MEHP, MEHHP, MEOHP and MECPP
# p<0.1; *p<0.05

Table S2. McCarthy Scales of Children's Abilities and individual geometric mean and sum of urinary levels of phthalate metabolites association by linear regression models, stratified by sex†

Metabolite	Motor Scale: Boys n = 93 ß† (95% CI)	Motor Scale: Girls n = 103 ß† (95% CI)	General Cognitive Index: Boys n = 93 ß† (95% CI)	General Cognitive Index: Girls n = 103 ß† (95% CI)
**∑ HMWP[1]**	-2.8 (-6.3, 0.8)	-2.5 (-5.5, 0.6)	-2.7 (-7.4, 2.0)	-2.5 (-6.9, 2.0)
**MEHP**	-1.4 (-4.2, 1.4)	-1.4 (-4.2, 1.5)	-1.4 (-5.2, 2.3)	-0.6 (-4.9, 3.5)
**MEHHP**	-2.3 (-5.3, 0.6)	-2.0 (-4.8, 0.6)	-1.6 (-5.7, 2.3)	-2.4 (-6.3, 1.5)
**MEOHP**	-2.2 (-5.3, 0.7)	-2.1 (-4.8, 0.6)	-2.0 (-6.0, 2.0)	-2.5 (-6.4, 1.4)
**MECPP**	-3.1 (-6.7, 0.6)	-2.2 (-5.2, 0.9)	-2.8 (-7.7, 2.0)	-1.9 (-6.3, 2.5)
**MBzP**	-0.9 (-3.4, 1.6)	-1.6 (-3.8, 0.5)	-2.0 (-5.2, 1.3)	-2.0 (-5.2, 1.1)
**MCPP**	-1.4 (-5.1, 2.1)	-0.4 (-3.1, 2.3)	-3.1 (-7.9, 1.6)	0.2 (-3.7, 4.1)
**∑ DEHP[2]**	-2.9 (-6.4, 0.6)	-2.3 (-5.4, 0.7)	-2.5 (-7.2, 2.1)	-2.3 (-6.7, 2.0)

† Adjusted for sex, gestational age, breastfeeding and maternal IQ
1 High Molecular Weight Phthalate Molar Sum: MEHP, MEHHP, MEOHP, MECPP, MBzP and MCPP.
2 Molar Sum of the four metabolites derived from DEHP: MEHP, MEHHP, MEOHP and MECPP
# p<0.1; *p<0.05

Table S3. Adjusted regression coefficients for change in McCarthy Scales of Children's Abilities associated with a 2nd trimester ln-unit increase in urinary phthalate metabolite concentration, stratified by sex

	Motor Scale		General Cognitive Index	
**Metabolite**	**Boys n = 75 ß† (95% CI)**	**Girls n = 93 ß† (95% CI)**	**Boys n = 75 ß† (95% CI)**	**Girls n = 93 ß† (95% CI)**
**∑ HMWP[1]**	-2.2 (-5.2,0.8)	-2.2 (-4.6,0.3)	-1.0 (-5.1,3.1)	-2.5 (-6.0,1.1)
**MEHP**	-1.9 (-4.8,0.9)	-0.8 (-3.2,1.5)	-1.4 (-5.2,2.5)	-1.7 (-5.1,1.6)
**MEHHP**	-1.7 (-4.3,0.8)	-1.8 (-3.9,0.3)	-0.8 (-4.2,2.6)	-2.2 (-5.2,-0.8)
**MEOHP**	-1.7 (-4.2,0.8)	-1.7 (-3.8,0.4)	-1.0 (-4.4,2.4)	-2.2 (-5.2,0.8)
**MECPP**	-2.4 (-5.8,1.0)	-2.0 (-4.5,0.6)	-0.6 (-5.2,4.0)	-2.1 (-5.8,1.6)
**MBzP**	-1.4 (-3.4,0.7)	-2.3* (-4.1,-0.5)	-1.6 (-4.3,1.1)	-1.6 (-4.3,1.1)
**MCPP**	0.2 (-3.0,3.5)	-0.4 (-2.9,2.1)	0.2 (-4.2,4.5)	1.1 (-2.5,4.7)
**∑ DEHP[2]**	-2.2 (-5.2,0.8)	-1.9 (-4.3,0.5)	-0.9 (-5.0,3.1)	-2.3 (-5.8,1.2)

† Adjusted for sex, gestational age, breastfeeding and maternal IQ
1 High Molecular Weight Phthalate Molar Sum: MEHP, MEHHP, MEOHP, MECPP, MBzP and MCPP.
2 Molar Sum of the four metabolites derived from DEHP: MEHP, MEHHP, MEOHP and MECPP
# p<0.1; *p<0.05

Table S4. Adjusted regression coefficients for change in McCarthy Scales of Children's Abilities associated with a 3rd trimester ln-unit increase in urinary phthalate metabolite concentration, stratified by sex.

	Motor Scale		General Cognitive Index	
**Metabolite**	**Boys n = 92 ß† (95% CI)**	**Girls n = 100 ß† (95% CI)**	**Boys n = 92 ß† (95% CI)**	**Girls n = 100 ß† (95% CI)**
**∑ HMWP[1]**	-0.4 (-3.2,2.4)	0.1 (-2.1,2.4)	-0.7 (-4.5,3.0)	-1.3 (-4.5,1.9)
**MEHP**	0.7 (-1.5,3.0)	0.9 (-1.2,3.0)	0.5 (-2.4,3.4)	0.6 (-2.4,3.7)
**MEHHP**	-0.5 (-2.9,1.8)	0.2 (-1.6,2.1)	-0.1 (-3.2,3.0)	-1.0 (-3.6,1.6)
**MEOHP**	-0.4 (-2.8,2.0)	0.1 (-1.8,1.9)	-0.5 (-3.7,2.7)	-1.0 (-3.6,1.7)
**MECPP**	-1.4 (-4.1,1.4)	0.1 (-1.9,2.1)	-1.6 (-5.3,2.1)	-0.8 (-3.7,2.0)
**MBzP**	1.6 (-0.7,3.8)	-0.3 (-2.3,1.6)	0.9 (-2.1,3.9)	-0.5 (-3.2,2.3)
**MCPP**	-0.8 (-3.3,1.8)	0.3 (-1.9,2.5)	-2.3 (-5.6,1.0)	-1.0 (-4.1,2.0)
**∑ DEHP[2]**	-0.7 (-3.4,2.0)	0.2 (-1.9,2.3)	-0.7 (-4.4,2.9)	-1.1 (-4.1,1.9)

† Adjusted for sex, gestational age, breastfeeding and maternal IQ
1 High Molecular Weight Phthalate Molar Sum: MEHP, MEHHP, MEOHP, MECPP, MBzP and MCPP.
2 Molar Sum of the four metabolites derived from DEHP: MEHP, MEHHP, MEOHP and MECPP
# p<0.1; *p<0.05

Table S5. Median urinary phthalate metabolites levels during pregnancy, measured in different studies.

Metabolite (ng/L) MEHP	MBzP	MCPP	Country	Sampling year	Number of participants	Reference
7.5	5.2	1.5	Mexico	1997- 2005	218	This study
16.7	10.1	6.1	France	2007	279	FA Zernana et al (2013)
4.6	12.1	-	USA	2000	25	Adibi et al. (2003)
3.3	8.3	2.1	USA	1999-2002	214	Swan et al. (2005)
6.0	22.0	3.2	USA	1998-2002	404	Wolff et al. (2008)
6.9	7.5	1.0	Netherlands	2004-2006	99	Yan et al. (2008)
6.8	5.3	1.3	Israel	2006	19	Berman et al. (2009)
4.0	3.7	-	Japan	2005-2006	50	Suzuki et al. (2009)
20.6	0.9	-	Taiwan	2005-2006	76	Huang et al. (2007)
19.2	1.9	1.2	Brazil	2012-2013	300	BA Rocha et al (2017)
